# Lipid emulsion inhibits the vasodilation induced by a toxic dose of amlodipine in isolated rat aortae

**DOI:** 10.7150/ijms.38502

**Published:** 2019-11-09

**Authors:** Seong-Ho Ok, Soo Hee Lee, Ji-Yoon Kim, Hyun-Jin Kim, Sung Il Bae, Yeran Hwang, Seongyeong Tak, Ju-Tae Sohn

**Affiliations:** 1 Department of Anesthesiology and Pain Medicine, Gyeongsang National University Changwon Hospital, Changwon, 51427, Republic of Korea;; 2 Department of Anesthesiology and Pain Medicine, Gyeongsang National University College of Medicine, Gyeongsang National University Hospital, 15 Jinju-daero 816 beon-gil, Jinju-si, Gyeongsangnam-do, 52727, Republic of Korea;; 3 Department of Anesthesiology and Pain Medicine, Gyeongsang National University Hospital, 15 Jinju-daero 816 beon-gil, Jinju-si, Gyeongsangnam-do, 52727, Republic of Korea;; 4 Division of Applied Life Sciences (BK21 plus), Gyeongsang National University, Gyeongsang, Republic of Korea;; 5 Department of Food Science & Technology, Institute of Agriculture and Life Science, Gyeongsang National University, Gyeongsang, Republic of Korea;; 6 Institute of Health Sciences, Gyeongsang National University, Jinju-si, 52727, Republic of Korea.

**Keywords:** amlodipine, lipid emulsion, centrifuged aqueous extract, vasodilation, verapamil

## Abstract

The goal of this study was to examine the effect of lipid emulsion on the vasodilation induced in isolated endothelium-denuded rat aortae by a toxic dose of amlodipine. We examined the effects of lipid emulsion and verapamil on amlodipine-induced vasodilation. We also examined the effects of a mixture of lipid emulsion and amlodipine, as well as the centrifuged aqueous extract (CAE) obtained by ultracentrifuging such a mixture and then removing the upper lipid layer, on amlodipine-induced vasodilation. The effect of lipid emulsion on the amlodipine concentration was examined. Lipid emulsion attenuated amlodipine-induced vasodilation in isolated aortae. Both CAE and lipid emulsion containing amlodipine inhibited amlodipine-induced vasodilation. However, there was no significant difference in amlodipine-induced vasodilation between aortae treated with CAE and those treated with lipid emulsion containing amlodipine. Verapamil inhibited amlodipine-induced vasodilation. Lipid emulsion decreased the concentration of amlodipine. Lipid emulsion attenuated the vasodilation induced by a toxic amlodipine dose in NaF-precontracted aortae. The data show that lipid emulsion inhibited the vasodilation induced by a toxic amlodipine dose in isolated rat aortae by reducing the concentration of amlodipine. Amlodipine-induced vasodilation seems to be mediated mainly by blockade of L-type calcium channels and partially by inhibition of the Rho-kinase pathway.

## Introduction

Clinical case reports and reviews describe the effective use of lipid emulsion in treating cardiovascular depression and cardiac arrhythmia induced by toxic doses of local anesthetics or other drugs with high lipid solubility 1-3. Lipid emulsion alleviates cardiovascular collapse induced by toxic doses of calcium channel blockers, such as verapamil 4-7. In addition, lipid emulsion can treat severe intractable cardiovascular depression caused by toxic doses of amlodipine, which is a dihydropyridine L-type calcium channel blocker and an antihypertensive drug 8,9. The proposed underlying mechanism of lipid emulsion treatment includes the scavenging effect, the inotropic effect, fatty acid supply, the attenuation of mitochondrial dysfunction, the inhibition of nitric oxide release and the attenuation of sodium channel blockade 1,10. The scavenging effect is widely accepted to be among the underlying mechanisms associated with the lipid emulsion treatment of local anesthetic systemic toxicity 10,11. This scavenging effect, which involves lipid sinks and shuttles, implies that highly lipid-soluble drugs (log [octanol/water partition coefficient]: > 2) are absorbed into the lipid phase of lipid emulsions and then transported into detoxifying and storage organs such as the liver and muscles 10,11. Centrifuged aqueous extract (CAE), the lower layer obtained after ultracentrifugation of a mixture of lipid emulsion and the highly lipid-soluble local anesthetic bupivacaine, attenuates the decreased blood pressure and sodium channel blockade induced by bupivacaine alone 12,13. In the case of vasodilation induced by a toxic dose of calcium channel blockers, the upper lipid layer produced by ultracentrifugation of the mixture of the calcium channel blocker and lipid emulsion seems to sequester a greater proportion of a lipid-soluble drug (verapamil) than of a less lipid-soluble drug (diltiazem), which decreases the amount of verapamil in the CAE and consequently the vasodilation induced by CAE compared with verapamil alone in Krebs solution 14-16. However, the effect of lipid emulsion on the vasodilation induced by a toxic dose of amlodipine remains unknown. Therefore, based on previous reports, as amlodipine is relatively lipid soluble (log [octanol/water partition coefficient]: 3.0) and a calcium channel blocker, we hypothesized that lipid emulsion inhibits the vasodilation induced by a toxic dose of amlodipine by decreasing the concentration of the drug 14-17. The objectives of this study were to examine the effect of lipid emulsion (Intralipid), which contains 100% long-chain fatty acid, on the vasodilation induced by a toxic dose of amlodipine in isolated rat aortae and to investigate its underlying mechanism and the cellular signaling pathway of amlodipine-induced vasodilation.

## Materials and Methods

Approval for all the experimental protocols was obtained from the Institutional Animal Care and Use Committee of Gyeongsang National University. All the experimental procedures and protocols were conducted according to the Guide for the Care and Use of Laboratory Animals stipulated by the Institute for Laboratory Animal Research.

### Preparation of isolated rat aortae followed by isometric tension measurement

Isolated rat thoracic aortae were prepared for measurements of isometric tension as previously described by our laboratory 18. Carbon dioxide (100%) was used to euthanize male Sprague-Dawley rats (body weight: 250-300 g). The descending thoracic aorta of each rat was dissected out and removed from the thorax. The isolated descending thoracic aorta was bathed in Krebs solution. Krebs solution contains the following components (mM): sodium chloride (118), glucose (11), sodium bicarbonate (25), potassium chloride (4.7), calcium chloride (2.4), magnesium sulfate (1.2) and monopotassium phosphate (1.2). The periaortic tissues containing connective tissue and fat were dissected and removed under a microscope. The aorta was cut into an aortic ring approximately 2.5 mm long. The endothelium of the aortic ring was removed by inserting 25-gauge needles into the lumen of the isolated aorta and rolling the vessel back and forth for a few seconds. Isolated aortic rings were suspended in a Grass isometric transducer (FT-03, Grass Instruments, Quincy, MA, USA) attached to an organ bath containing 10 mL of Krebs solution maintained at 37°C. A baseline resting tension of 3.0 g was maintained for 120 min to reach equilibrium, and the Krebs solution was exchanged approximately every 30 min 18,19. A mix of 95% oxygen gas and 5% carbon dioxide gas was supplied to aerate the Krebs solution in the organ bath to maintain a pH of approximately 7.4. To confirm endothelial denudation of the isolated rat aortae, we added phenylephrine (10^-8^ M) into the organ bath containing the aortae. After phenylephrine (10^-8^ M) produced a sustained and stable concentration, we added acetylcholine (10^-5^ M) into the organ bath to verify endothelial denudation; any aorta that relaxed by less than 15% in response to acetylcholine was considered to be denuded of its endothelium. After the aortic rings showing acetylcholine-induced relaxation were washed with fresh Krebs solution, baseline resting tension was recovered. Then, contraction of some isolated endothelium-denuded rat aortic rings was induced by isotonic 60 mM KCl, and the magnitude of the resulting contraction was used as a reference value to express the magnitude of norepinephrine-induced contraction. Next, the isotonic 60 mM KCl solution was washed away with fresh Krebs solution, and baseline resting tension was restored. Then, we performed the following experimental protocols. As amlodipine induces the nitric oxide-dependent relaxation of isolated aorta and lipid emulsion attenuates acetylcholine-induced nitric oxide-dependent relaxation, the isolated endothelium-denuded aortic rings used for the following experimental protocols were pretreated with the nitric oxide synthase inhibitor N^W^-nitro-L-arginine methyl ester (L-NAME, 10^-4^ M) to avoid the confounding factor of nitric oxide release from any residual endothelium; the abovementioned two factors hypothetically might complicate the interpretation of results regarding the effect of lipid emulsion on amlodipine-induced vasodilation in the current study 20,21.

### Experimental protocol

First, we examined the effect of cotreatment with lipid emulsion on the vasodilation induced by toxic concentrations of amlodipine in isolated endothelium-denuded rat aortae precontracted with norepinephrine. Norepinephrine (10^-6^ M) was added to the organ bath to produce sustained and stable vasoconstriction. Then, a toxic dose of amlodipine (3 × 10^-7^ M) alone or combined with lipid emulsion (0.25 and 1%) was added to produce vasodilation in isolated endothelium-denuded rat aortae precontracted with norepinephrine 22. Amlodipine-induced vasodilation was monitored for 70 min in the presence and absence of lipid emulsion. In addition, we examined the effect of posttreatment with lipid emulsion on the vasodilation induced by amlodipine in the endothelium-denuded rat aorta precontracted with KCl. After contraction induced by isotonic 60 mM KCl solution reached a plateau in the aortic rings, amlodipine (10^-7^ M) was added to the organ bath. After amlodipine-induced vasodilation reached approximately 20% vasodilation with respect to the previous contraction induced by the KCl solution, some aortic rings were treated with lipid emulsion (0.3 and 1%). Then, amlodipine-induced vasodilation was monitored for 70 min in the presence or absence of lipid emulsion. Additionally, we compared the magnitude of amlodipine-induced vasodilation for 70 min in isolated rat aorta precontracted with norepinephrine (10^-6^ M) or 60 mM KCl.

**Figure 1:**
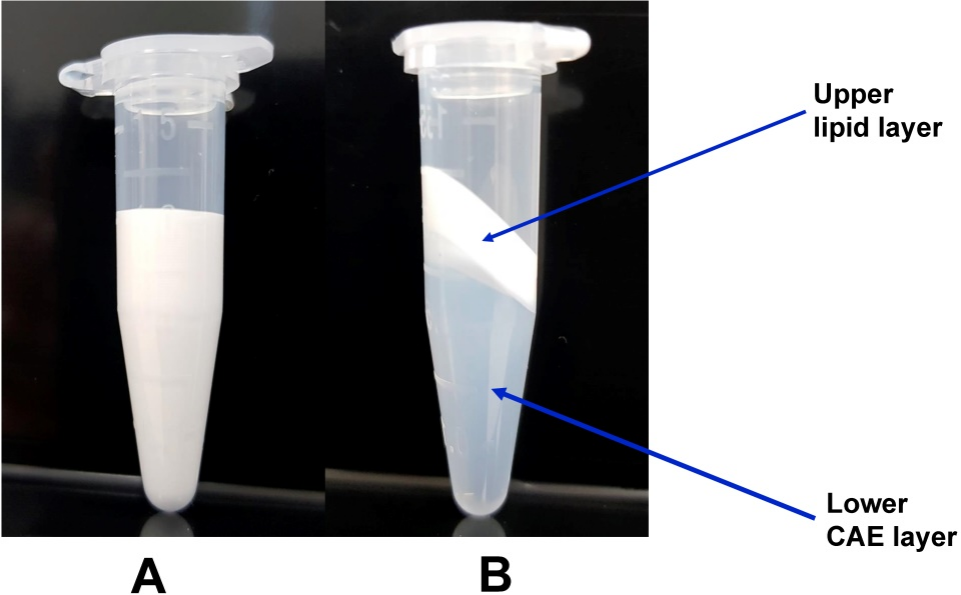
** This figure shows the conditions before (A)** and after **(B)** ultracentrifugation (75,000 *g* at 4°C for 12 min) of approximately 20% Intralipid containing approximately 6 **×** 10^-6^ M amlodipine. Ultracentrifugation separated the mixture into the upper lipid layer and lower centrifuged aqueous extract (CAE) layer.

Second, based on a previous report that lipid emulsion attenuates the vasodilation induced by toxic doses of highly lipid-soluble calcium channel blockers (bepridil and verapamil) by reducing the concentrations of these drugs in the CAE because larger amounts of these drugs partition into the upper lipid layer compared with the less lipid-soluble calcium channel blocker diltiazem, we examined the effects of a mixture of lipid emulsion and amlodipine as well as CAE obtained by ultracentrifuging such a mixture on isolated endothelium-denuded rat aortae precontracted with norepinephrine 16. CAE, which is used in this experimental protocol, was obtained from the ultracentrifugation of the mixture of lipid emulsion plus amlodipine and the subsequent removal of the upper lipid layer (Fig. 1). After norepinephrine (10^-6^ M) produced a sustained and stable contraction, CAE or amlodipine with or without lipid emulsion was added to the organ bath to produce amlodipine-induced vasodilation in the precontracted aortae, and then amlodipine-induced vasodilation was monitored for 70 min. Lipid emulsion containing amlodipine and CAE were produced using the following methods. Lipid emulsion (20%) 10 mL and amlodipine (6 × 10^-3^ M) 10 µL were mixed using a Mylab Multi Mixer (#SLRM-3, Seoulin Bioscience, Bundang, Korea) at 60 RPM for 30 min to produce approximately 20% lipid emulsion containing approximately 6 × 10^-6^ M amlodipine (Fig. 1A). A 500 µL volume of the resulting mixture was added to the organ bath with 9.5 mL Krebs solution so that the bath contained 1% lipid emulsion and 3 × 10^-7^ M amlodipine. Approximately 20% lipid emulsion containing approximately 6 × 10^-6^ M amlodipine was ultracentrifuged at 4°C and 75,000 *g* for 12 min using an Optima^TM^ MAX-XP ultracentrifuge (Beckman Coulter Life Sciences, Indianapolis, IN, USA) to separate the emulsion into two layers: a lower CAE layer (Fig. 1B) and an upper lipid layer (Fig. 1B). Five hundred microliters of this lower layer (Fig. 1B) was added to the organ bath with 9.5 mL Krebs solution. The resulting CAE corresponded to 1% lipid emulsion and 3 × 10^-7^ M amlodipine in the organ bath. A 490 µL volume of Krebs solution and a 10 µL volume of amlodipine (3 × 10^-4^ M) were added to the organ bath with 9.5 mL Krebs solution so that the amlodipine (at a final concentration of 3 × 10^-7^ M) would induce vasodilation. We examined the effect of pretreatment with verapamil on amlodipine-induced vasodilation in isolated endothelium-denuded rat aortae to confirm that amlodipine-induced vasodilation involves the blockade of L-type calcium channels 23. After endothelium-denuded rat aortae were pretreated with verapamil (10^-5^ M) for 20 min, norepinephrine (10^-6^ M) was added to the organ bath to produce vasoconstriction in the presence or absence of verapamil. Then, amlodipine (3 × 10^-7^ M) was added to the organ bath to produce vasodilation in the presence or absence of verapamil. For comparison with amlodipine-induced vasodilation in the presence of verapamil, some endothelium-denuded aortic rings were not posttreated with amlodipine, and vasodilation was induced by verapamil alone (10^-5^ M). The vasodilation induced by amlodipine or verapamil alone and combined treatment with verapamil and amlodipine was monitored for 70 min and compared.

Third, we examined the effects of the Rho-kinase inhibitor Y-27632 (10^-6^ to 10^-5^ M) and the protein kinase C (PKC) inhibitor GF109203X (10^-6^ to 10^-5^ M) on the contraction induced by norepinephrine (10^-9^ to 10^-5^ M) in isolated endothelium-denuded rat aortae. Isolated endothelium-denuded rat aortic rings were pretreated with Y-27632 (10^-6^ to 10^-5^ M) and GF109203X (10^-6^ to 10^-5^ M) for 20 min. Then, norepinephrine (10^-9^ to 10^-5^ M) was cumulatively added to the organ bath to produce a norepinephrine concentration-response curve in the presence or absence of Y-27632 or GF109203X.

Fourth, as the precontraction induced by norepinephrine involves the PKC- and Rho-kinase-mediated pathway, as demonstrated by the results of the experimental protocol above, we examined the effect of pretreatment with lipid emulsion on the vasodilation induced by amlodipine in isolated endothelium-denuded rat aortae precontracted with the Rho-kinase activator sodium fluoride (NaF) or the PKC activator phorbol 12,13-dibutyrate (PDBu) 23. Endothelium-denuded rat aortae were pretreated with lipid emulsion (0.25 and 1%) for 20 min. Then, NaF (3 × 10^-8^ M) or PDBu (10^-6^ M) was added to the organ bath to produce a sustained and stable contraction in the presence or absence of lipid emulsion. Then, amlodipine (3 × 10^-7^ M) was added to the organ bath to produce vasodilation. Amlodipine-induced vasodilation was monitored for 70 min. In addition, we compared the effects of NaF and PDBu on amlodipine (3 × 10^-7^ M)-induced vasodilation for 70 min to investigate the underlying mechanism of the amlodipine-induced vasodilation of isolated aortae.

### Effect of lipid emulsion on the amlodipine concentration in Krebs solution

Amlodipine (3 × 10^-7^ M) in Krebs solution was mixed with Intralipid (0.25 and 1%) with a rotator for 30 min to emulsify the amlodipine and lipid emulsion as previously described 16,24. After centrifugation at 75,000 *g* for 40 min, the amlodipine in the aqueous phase, which is equivalent to CAE, was measured by ultraperformance liquid chromatography-quadrupole time-of-flight mass spectrometry (UPLC-Q-TOF MS; Waters, Milford, MA, USA). The aqueous layer was injected into an Acquity UPLC BEH C_18_ column (100 × 2.1 mm, 1.7 µm; Waters) equilibrated with water/acetonitrile (95:5) containing 0.1% formic acid and eluted with a linear gradient (5-100%) of acetonitrile containing 0.1% formic acid at a flow rate of 0.35 mL/min for 7 min. The eluted amlodipine was analyzed by Q-TOF MS (Waters) in positive electrospray ionization (ESI) mode and identified by the precursor and product ions of m/z 289.21 and 140.13, respectively, in multiple reaction monitoring. The capillary and sampling cone voltages were set at 3 kV and 30 V, respectively. The desolvation temperature and flow rate were 100°C and 800 L/h, respectively. The source temperature was set at 400°C. LockSpray with leucine-enkephalin ([M+H]=556.2771 Da) was used at a frequency of 10 seconds to ensure the reproducibility and accuracy of all analyses. All mass data were collected and analyzed by UIFI 1.8.2 (Waters).

### Materials

Norepinephrine, KCl, amlodipine, PDBu, NaF, GF109203X, L-NAME, verapamil, phenylephrine and acetylcholine were obtained from Sigma Aldrich (St. Louis, MO, USA). Y-27632 was obtained from Calbiochem (La Jolla, CA, USA). Intralipid^®^ (20%) was obtained from Fresenius Kabi Korea (Seoul, Korea). All chemical concentrations are expressed as the final molar concentration in the organ bath. The concentration of lipid emulsion is expressed as the percentage in the organ bath. PDBu, GF109203X and amlodipine were dissolved in dimethyl sulfoxide (final concentration of dimethyl sulfoxide: less than 0.1%). All the chemicals except PDBu, GF109203X and amlodipine were dissolved in distilled water.

### Statistical analysis

Data are expressed as the mean ± SD or median ± interquartile range. The vasodilation induced by amlodipine is expressed as the percentage of contraction induced by norepinephrine, KCl, PDBu and NaF. The effects of lipid emulsion, CAE and verapamil on amlodipine-induced vasodilation were analyzed using a generalized linear mixed-effects model (Stata version 14.2, StataCorp LP, Lakeway Drive College Station, TX, USA) 25. The magnitude of norepinephrine-induced contraction is expressed as the percentage of maximal contraction induced by isotonic 60 mM KCl. The effects of Y-27632 and GF109203X on norepinephrine-induced contraction and the effects of NaF, PDBu, norepinephrine and KCl on amlodipine-induced vasodilation were analyzed with a generalized linear mixed-effects model. The effect of lipid emulsion on amlodipine concentration was analyzed using Kruskal-Wallis test followed by Dunn's multiple comparison test. A *P* value less than 0.05 was considered statistically significant.

## Results

**Figure 2:**
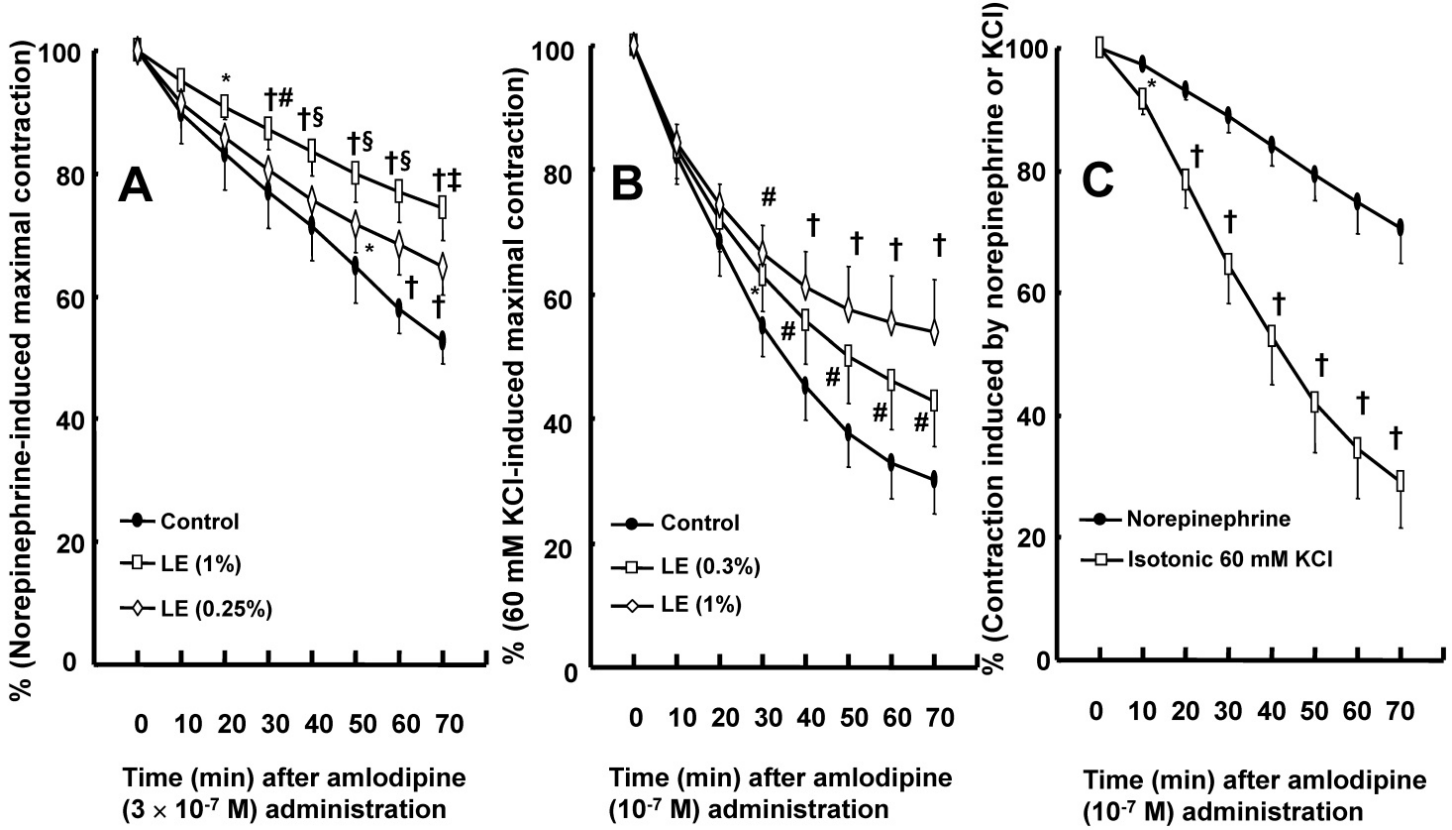
** A:** Effect of cotreatment with lipid emulsion (Intralipid, LE) on the vasodilation induced by amlodipine (3 **×** 10^-7^ M) in isolated endothelium-denuded rat aortae precontracted with norepinephrine (10^-6^ M). Data (N = 6) are shown as the mean ± SD and expressed as the percentage of maximal contraction induced by norepinephrine. N represents the number of isolated aortae. *P < 0.01 and †*P* < 0.001 versus control. #*P*< 0.05, §*P* < 0.01 and ‡*P* < 0.001 versus 0.25% LE. **B:** Effect of posttreatment with LE on the vasodilation induced by amlodipine (10^-7^ M) in isolated endothelium-denuded rat aortae precontracted with 60 mM KCl. After amlodipine (10^-7^ M) produced approximately 20% vasodilation from the contraction induced by isotonic 60 mM KCl, some aortic rings were treated with LE (0.3 and 1%). Data (control and 1% LE: N = 5; 0.3% LE: N = 4) are shown as the mean ± SD and expressed as the percentage of contraction induced by isotonic 60 mM KCl. N represents the number of rats from which isolated aortae were obtained. *P < 0.05, #*P* <0.01 and †*P* < 0.001 versus control. **C:** Amlodipine-induced vasodilation in isolated endothelium-denuded rat aortae precontracted with norepinephrine (10^-6^ M) or isotonic 60 mM KCl. Data (N = 8) are shown as the mean ± SD and expressed as the percentage of contraction induced by KCl or norepinephrine. N represents the number of isolated rat aortae. *P < 0.05 and †*P* < 0.001 versus norepinephrine.

Cotreatment with lipid emulsion (0.25 and 1%) inhibited amlodipine (3 × 10^-7^ M)-induced vasodilation in isolated endothelium-denuded rat aortae precontracted with norepinephrine in a concentration-dependent pattern (Fig. 2A; *P* < 0.01 versus control at 50 and 70 min). Posttreatment with lipid emulsion (0.3 and 1%) attenuated amlodipine (10^-7^ M)-induced vasodilation in isolated endothelium-denuded rat aortae precontracted with isotonic 60 mM KCl (Fig. 2B; *P* < 0.05 versus control at 30 to 70 min). Amlodipine (10^-7^ M)-induced vasodilation was enhanced under KCl-induced contraction compared with norepinephrine-induced contraction (Fig. 2C; *P* < 0.05 versus 10 to 70 min).

**Figure 3:**
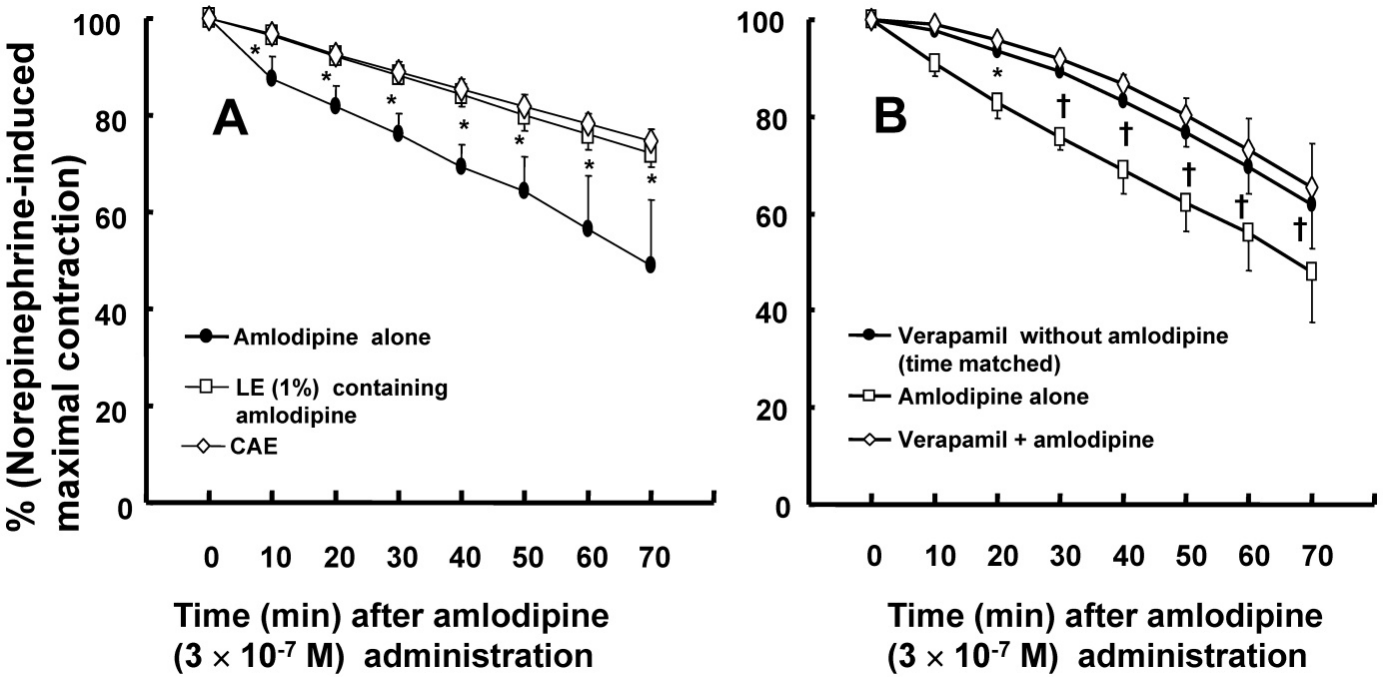
** A:** Effects of a mixture of lipid emulsion (Intralipid, LE: 1%) and amlodipine (3 **×** 10^-7^ M), centrifuged aqueous extract (CAE) and amlodipine (3 **×** 10^-7^ M) alone in isolated endothelium-denuded rat aortae precontracted with norepinephrine (10^-6^ M). The CAE, which was obtained by using ultracentrifugation to separate the lipid emulsion containing amlodipine into an upper lipid layer and a lower CAE layer, corresponds to LE (1%) and amlodipine (3 **×** 10^-7^ M) in the organ bath. Data (N = 9) are shown as the mean ± SD and expressed as the percentage of maximal contraction induced by norepinephrine. N represents the number of isolated rat aortae. *P < 0.001 versus amlodipine alone. **B:** The vasodilation induced by amlodipine or verapamil alone and by combined treatment with verapamil and amlodipine in isolated endothelium-denuded rat aorta precontracted with norepinephrine (10^-6^ M). Data (verapamil without amlodipine: N = 8, amlodipine alone: N = 5, and verapamil plus amlodipine: N = 8) are shown as the mean ± SD and expressed as the percentage of maximal contraction induced by norepinephrine. N represents the number of isolated rat aortae. *P < 0.01 and †*P* < 0.001 versus amlodipine alone.

Both CAE, which corresponds to a 1% lipid emulsion containing 3 × 10^-7^ M amlodipine in the organ bath, and lipid emulsion (1%) containing amlodipine (3 × 10^-7^ M) inhibited amlodipine-induced vasodilation (Fig. 3A; *P* < 0.001 versus amlodipine at 10 to 70 min). However, CAE did not significantly inhibit amlodipine-induced vasodilation compared with lipid emulsion (1%) containing amlodipine (3 × 10^-7^ M) (Fig. 3A). Pretreatment with verapamil (10^-5^ M) inhibited amlodipine (3 × 10^-7^ M)-induced vasodilation in isolated endothelium-denuded rat aortae precontracted with norepinephrine (Fig. 3B; *P* < 0.01 versus amlodipine alone at 20 to 70 min). However, there was no significant difference between vasodilation induced by verapamil (10^-5^ M) alone and vasodilation induced by verapamil (10^-5^ M) plus amlodipine (3 × 10^-7^ M) in isolated rat aortae precontracted with norepinephrine (Fig. 3B).

**Figure 4:**
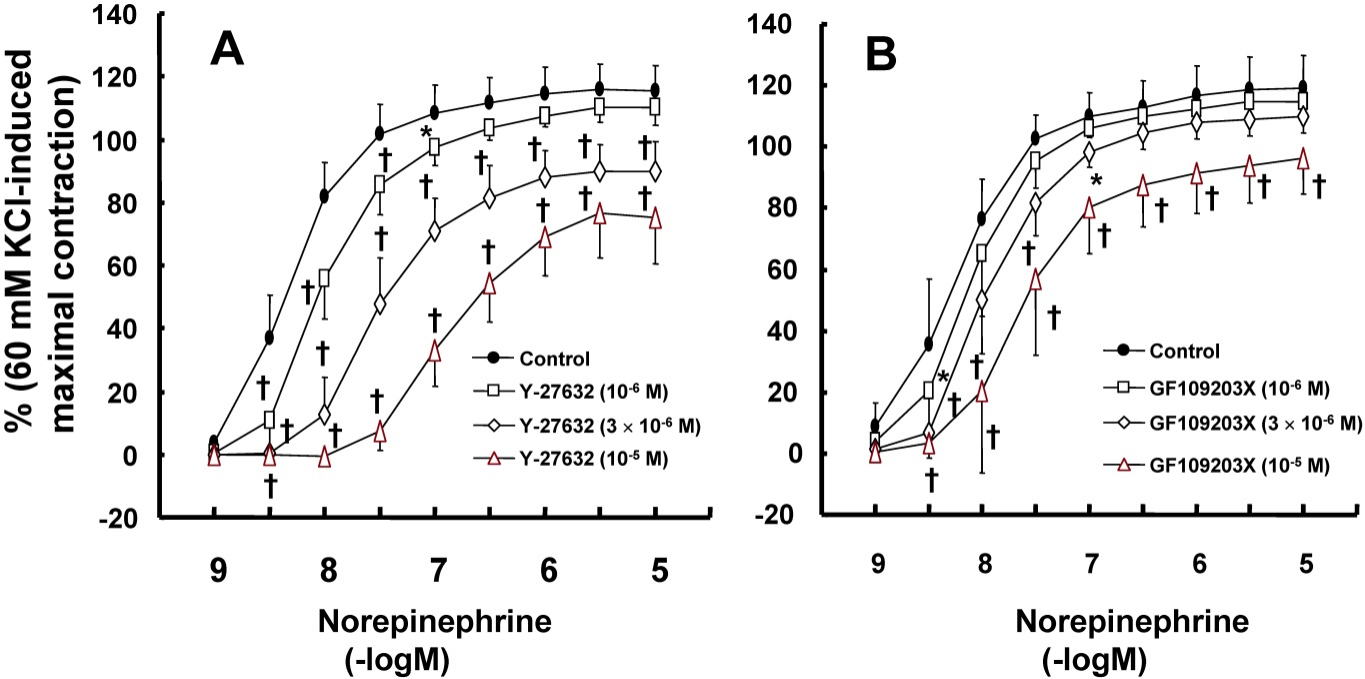
Effects of Rho-kinase inhibitor Y-27632 (**A**; N = 8) and protein kinase C inhibitor GF109203X (**B**; control: N = 8, 10^-6^ M: N = 7; 3 **×** 10^-6^ and 10^-5^ M = 8) on the contraction induced by norepinephrine in isolated endothelium-denuded rat aortae. Data are shown as the mean ± SD and expressed as the percentage of maximal contraction induced by 60 mM KCl. N represents the number of isolated rat aortae. *P < 0.05 and †*P* < 0.001 versus control.

Y-27632 (10^-6^ to 10^-5^ M) inhibited norepinephrine-induced contraction (Fig. 4A; 3 × 10^-6^ and 10^-5^ M: *P* < 0.001 versus control at 3 × 10^-9^ to 10^-5^ M norepinephrine), as did GF109203X (3 × 10^-6^ and 10^-5^ M Fig. 4B; *P* < 0.001 versus control at 3 × 10^-9^ to 3 × 10^-8^ M norepinephrine).

**Figure 5:**
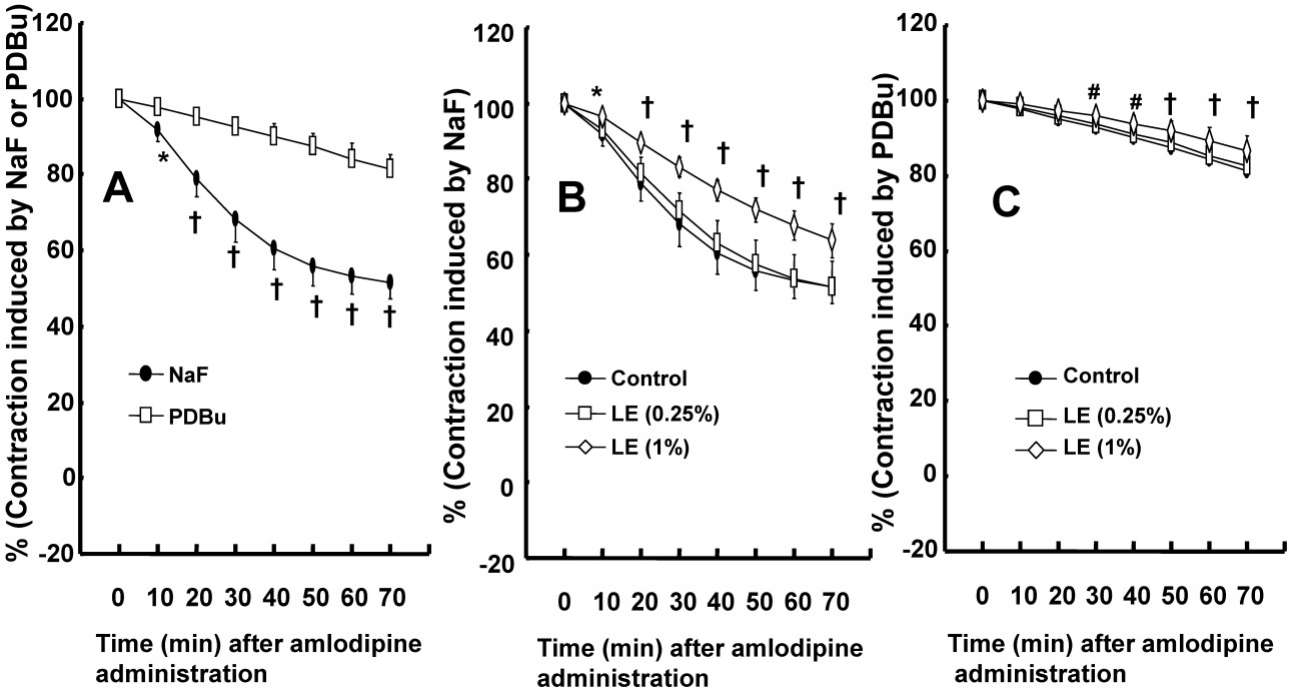
** A:** Amlodipine (3 **×** 10^-7^ M)-induced vasodilation in isolated endothelium-denuded rat aortae precontracted with the Rho-kinase activator sodium fluoride (NaF, 8 **×** 10^-3^ M) or the protein kinase C activator phorbol 12,13-dibutyrate (PDBu, 10^-6^ M). Data (N = 8) are shown as the mean ± SD and expressed as the percentage of maximal contraction induced by NaF or PDBu. N represents the number of isolated rat aortae. *P < 0.05 and †*P* < 0.001 versus PDBu. **B** and **C**: Effect of pretreatment with lipid emulsion (Intralipid, LE) on the vasodilation induced by amlodipine (3 **×** 10^-7^ M) in isolated endothelium-denuded rat aorta precontracted with NaF (8 **×** 10^-3^ M, B) or PDBu (10^-6^ M, C). Data (N = 8) are shown as the mean ± SD and expressed as the percentage of maximal contraction induced by NaF or PDBu. N represents the number of isolated rat aortae. *P < 0.05, #*P* <0.01 and †*P* < 0.001 versus control.

Amlodipine (3 × 10^-7^ M)-induced vasodilation was higher in endothelium-denuded rat aorta precontracted with NaF (8 × 10^-3^ M) than in those precontracted with PDBu (10^-6^ M) (Fig. 5A; *P* < 0.05 versus PDBu at 10 to 70 min). Lipid emulsion (1%) attenuated amlodipine (3 × 10^-7^ M)-induced vasodilation in isolated endothelium-denuded rat aortae precontracted with NaF (Fig. 5B; *P* < 0.05 versus control at 10 to 70 min). However, lipid emulsion (1%) slightly inhibited amlodipine (3 × 10^-7^ M)-induced vasodilation in isolated endothelium-denuded rat aortae precontracted with PDBu (Fig. 5C; *P* < 0.01 versus control at 30 to 70 min).

**Figure 6:**
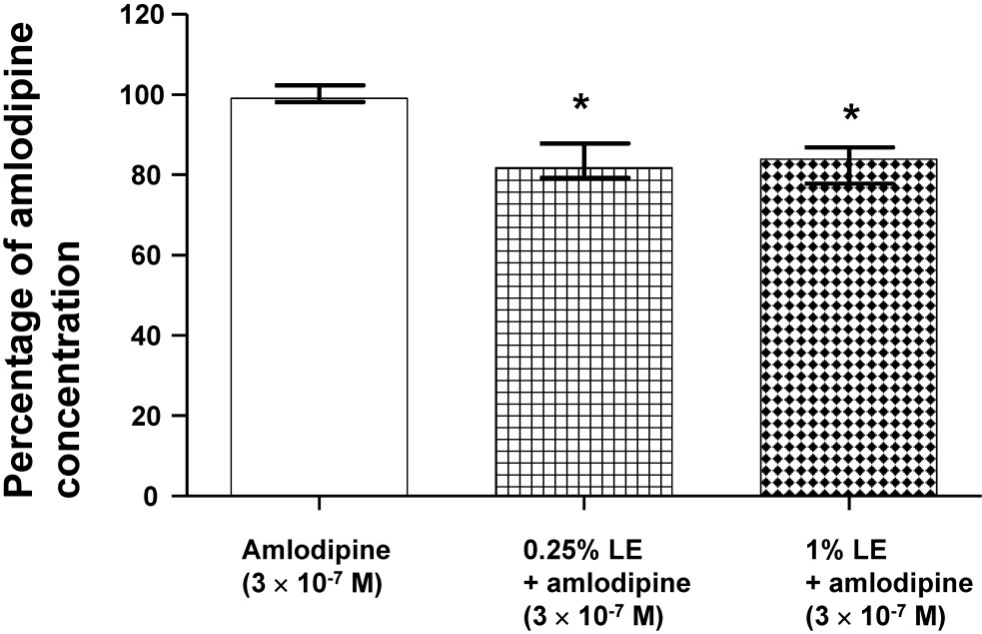
Effect of lipid emulsion (Intralipid, LE) on the amlodipine concentration (3 **×** 10^-7^ M) in Krebs solution. Amlodipine concentration was measured using ultraperformance liquid chromatography-quadrupole time-of-flight mass spectrometry. Data are shown as the median ± interquartile range and expressed as the percentage of the original amlodipine concentration (3 **×** 10^-7^ M). Statistical analysis was performed using the Kruskal-Wallis test followed by Dunn's multiple comparison test. Experiments were repeated five times. *P < 0.05 versus amlodipine alone.

Lipid emulsion (0.25 and 1%) significantly decreased the concentration of amlodipine (originally 3 × 10^-7^ M; Fig. 6; *P* < 0.05 versus amlodipine alone) in CAE, which was obtained by ultracentrifugation of the mixture of lipid emulsion and amlodipine.

## Discussion

This study suggests that lipid emulsion attenuates the vasodilation induced by a toxic dose of amlodipine, which is associated with the reduction in amlodipine concentrations caused by lipid emulsion. The major findings of this study are as follows: 1) cotreatment or posttreatment with lipid emulsion attenuated the amlodipine-induced vasodilation of isolated aortae; 2) CAE produced less vasodilation than amlodipine alone; 3) there was no significant difference between vasodilation induced by verapamil alone and vasodilation induced by combined treatment with verapamil and amlodipine; and 4) the concentration of amlodipine was decreased in the CAE (Fig. 6), which was derived from the mixture of lipid emulsion and amlodipine.

The treatment of toxicity induced by calcium channel blockers includes gastrointestinal decontamination, supportive care, vasopressors, calcium, hyperinsulinemia/euglycemia, lipid emulsion and extracorporeal membrane oxygenation 26. Among these treatments, lipid emulsion and extracorporeal membrane oxygenation can be used to treat intractable hypotension induced by a toxic dose of amlodipine 8,9,26,27. The lipid sink theory, which is popularly accepted, indicates that highly lipid-soluble drugs are absorbed into lipid emulsion from tissues including the heart and aorta 12,13,16,28-30. Lipid emulsion inhibits the vasodilatory effects of calcium channel blockers including, in decreasing order of inhibition, bepridil, verapamil and nifedipine; this phenomenon seems to be associated with lipid solubility 14. In addition, CAE, which is derived from a mixture of lipid emulsion and a relatively lipid-soluble calcium channel blocker (bepridil or verapamil) by ultracentrifugation of the mixture followed by the removal of the upper lipid layer, attenuated vasodilation induced by toxic doses of bepridil and verapamil alone but did not significantly inhibit vasodilation induced by bepridil or verapamil alone compared with a mixture of lipid emulsion and a calcium channel blocker 16. Moreover, CAE from a mixture of the highly lipid-soluble local anesthetic bupivacaine and lipid emulsion produces a smaller decrease in mean blood pressure than bupivacaine alone 12. CAE obtained from the mixture of lipid emulsion and bupivacaine partially reverses the sodium current blockade induced by bupivacaine alone 13. These previous studies suggest that when a lower CAE layer and an upper lipid layer from the mixture of lipid emulsion with lipid-soluble drugs are separated by ultracentrifugation (Fig. 1B), a large portion of relatively lipid-soluble drugs (bepridil, verapamil and bupivacaine) is sequestered into the upper lipid layer, leading to reduced concentrations of relatively lipid-soluble drugs in the lower CAE layer and supporting the lipid sink theory 1,10,12,13,16. Similar to a previous report involving verapamil (log [octanol/water partition coefficient]: 3.79), lipid emulsion (0.25, 0.3 and 1%) attenuated the vasodilation caused by a toxic dose of amlodipine (Fig. 2A and B), and lipid emulsion (0.25 and 1%) decreased amlodipine (3 × 10^-7^ M) concentrations to 83 ± 6 and 82 ± 5% of their original concentrations, respectively (Fig. 6) 16,31. These findings suggest that this lipid emulsion-mediated attenuation of the vasodilation induced by a toxic dose of amlodipine is associated with the reduction in amlodipine concentration caused by lipid emulsion. The attenuation of amlodipine-induced vasodilation was greater at a high concentration (1%) of lipid emulsion than at a low concentration (0.25%) of lipid emulsion (Fig. 2A; *P* < 0.05 at 30 to 70 min). However, there was no significant difference in the reduction in amlodipine concentration by low (0.25%) and high (1%) concentrations of lipid emulsion (Fig. 6). Thus, there may be unidentified mechanisms other than a lipid sink associated with the lipid solubility of amlodipine. On the other hand, CAE and 1% lipid emulsion containing amlodipine did not significantly alter the reduction in amlodipine-induced vasodilation, suggesting that the lipid emulsion-mediated reduction in amlodipine-induced vasodilation may be mainly dependent on the lipid sink effect. This discrepancy (Fig. 2A, 3A and 6) may be due to physiochemical alterations in lipid emulsion containing amlodipine by the strong ultracentrifugation used to produce the lower CAE layer. As cardiovascular collapse induced by a toxic dose of amlodipine produces acidosis *in vivo*, further research on the effect of acidification on the lipid emulsion-mediated inhibition of vasodilation evoked by amlodipine is needed 32.

Amlodipine produces vasodilation in isolated rat aortae or pulmonary arteries precontracted with KCl and norepinephrine 33,34. Amlodipine-induced vasodilation is more enhanced in KCl-induced contraction than in norepinephrine-induced contraction 33. Consistent with previous reports, amlodipine induced vasodilation in rat aorta precontracted with norepinephrine or KCl (Fig. 2C), and the magnitude of vasodilation induced by amlodipine was higher in KCl-induced contraction than in norepinephrine-induced contraction (Fig. 2C) 33,34. The contraction induced by contractile agonists such as phenylephrine involves calcium influx via the L-type calcium channels 35. The vasodilation induced by verapamil alone was similar to that induced by combined treatment with verapamil and amlodipine (Fig. 3B), suggesting that the blockade of L-type calcium channels with verapamil abolishes amlodipine-induced vasodilation 33,35. In contrast to KCl-induced contraction, the contraction evoked by norepinephrine, which is released from sympathetic nerve endings, involves calcium sensitization-mediated contraction through the inhibition of myosin light chain phosphatase via Rho-kinase or PKC 36. Norepinephrine-induced contraction was inhibited by Rho-kinase inhibitor Y-27632 or PKC inhibitor GF109203X (Fig. 4A and B), showing that norepinephrine-induced contraction is mediated by a pathway involving PKC and Rho-kinase. As amlodipine-induced vasodilation was enhanced in contraction induced by the Rho-kinase stimulant NaF compared with the PKC stimulant PDBu (Fig. 5A), our findings as a whole indicate that the inhibition of amlodipine-induced vasodilation by lipid emulsion (1%) in isolated rat aortae precontracted with norepinephrine seems to rely partially on the maintenance of contraction mediated by Rho-kinase (Fig. 4A and 5A).

The limitations of this study are as follows: first, endothelial nitric oxide is involved in the regulation of vascular tone, whereas endothelium-denuded aorta pretreated with L-NAME was used in the current study to avoid compounding factors such as nitric oxide-dependent relaxation due to remaining endothelium after endothelial denudation 20,21. Second, small resistance arterioles are the main vessels involved in peripheral vascular resistance associated with blood pressure, whereas this experiment used aortae, which are considered conduit vessels 37. Third, a toxic dose of amlodipine causes myocardial depression, whereas this experiment did not consider the heart, which is important in controlling hemodynamics 33,38. Fourth, the triglyceride half-life of chylomicron, which transports lipids absorbed from the intestine, was reported to be approximately 5 to 6 min, whereas Intralipid, containing 100% long-chain triglycerides in aqueous Krebs solution, was used for approximately 70 min in the current experiment 39.

In conclusion, these results suggest that lipid emulsion inhibits the vasodilation induced by a toxic dose of amlodipine in isolated endothelium-denuded rat aorta by reducing the concentration of amlodipine. The vasodilation induced by a toxic dose of amlodipine in rat aortae precontracted with norepinephrine seems to be mediated mainly by blockade of the L-type calcium channel and partially by inhibition of the Rho-kinase-mediated pathway.

## Abbreviations

CAE: centrifuged aqueous extract
PKC: protein kinase C
